# Bayesian Analysis of Perceived Eye Level

**DOI:** 10.3389/fncom.2016.00135

**Published:** 2016-12-15

**Authors:** Elaine E. Orendorff, Laurynas Kalesinskas, Robert T. Palumbo, Mark V. Albert

**Affiliations:** ^1^École des Neurosciences de Paris, Université Pierre et Marie CurieParis, France; ^2^Department of Biology, Loyola University ChicagoChicago, IL, USA; ^3^Bioinformatics Program, Loyola University ChicagoChicago, IL, USA; ^4^Department of Psychology, Loyola University ChicagoChicago, IL, USA; ^5^Department of Medical and Social Sciences, Northwestern UniversityChicago, IL, USA; ^6^Department of Computer Science, Loyola University ChicagoChicago, IL, USA

**Keywords:** Bayesian analysis, perceived eye level, cue combination, elevation estimation, visual psychophysics

## Abstract

To accurately perceive the world, people must efficiently combine internal beliefs and external sensory cues. We introduce a Bayesian framework that explains the role of internal balance cues and visual stimuli on perceived eye level (PEL)—a self-reported measure of elevation angle. This framework provides a single, coherent model explaining a set of experimentally observed PEL over a range of experimental conditions. Further, it provides a parsimonious explanation for the additive effect of low fidelity cues as well as the averaging effect of high fidelity cues, as also found in other Bayesian cue combination psychophysical studies. Our model accurately estimates the PEL and explains the form of previous equations used in describing PEL behavior. Most importantly, the proposed Bayesian framework for PEL is more powerful than previous behavioral modeling; it permits behavioral estimation in a wider range of cue combination and perceptual studies than models previously reported.

## Introduction

On a daily basis, our bodies integrate millions of separate stimuli to enable our perception of the world, ranging from visual and auditory stimuli to internal cues. Our brain integrates all of these separate stimuli into what appear to be clear perceptions (Ernst, 2006). As a result, in order to fully understand perception, we must understand how various stimuli are combined and interpreted. Various methods have been used to accomplish this in previous research, such as maximum-likelihood estimation (Hillis et al., 2004), modified weak fusion (Landy et al., 1995), perturbation analysis (Young et al., 1993), and Bayesian analysis (Kersten et al., 2004). Importantly, all these behavioral modeling approaches use relatively simple mathematical frameworks to explain what can appear to be complex behavioral phenomena.

The use of Bayesian analysis in perceptual studies is well justified by previous models of perceptual decision making. The Bayesian coding hypothesis assumes that the brain represents information probabilistically (Knill and Pouget, 2004). Although, finding direct neural correlates of these statistical distributions is not always readily apparent, the ability to simulate behavior using these principles suggests this type of computation plays a substantial high-level role in perceptual processing. Previous research has explored this type of Bayesian analysis for cue combination, object perception (Kersten et al., 2004), spatial localization (Battaglia et al., 2003), and forms of visual perception (Bridgeman, 2003).

Perceived eye level (PEL) is a self-reported measure of elevation angle interpreted from a combination of visual and internal cues. Estimating PEL is important as having an accurate PEL plays a role in judging height, distance, elevation, and size of objects. Different factors can confound our perception of eye level, however, causing PEL to differ from the actual eye level. These factors can be classified as internal factors, such as the head's position relative to gravity, or external factors, such as strength of visual stimuli. Further, it is known that visual stimuli can contribute to the error in perceiving eye level as we make a wide variety of assumptions regarding the orientation of objects relative to gravity. For example, we have a strong disposition to assume near-vertical lines on a distant wall are truly vertical, and observed visual pitches can be attributed to perspective above or below the horizontal. Estimating this behavior can be structured as a Bayesian cue-combination problem, modeling the effect of multiple stimuli on the perception of eye level.

In this study, we use Bayesian analysis to derive a simple framework that can be used to estimate perception from multiple sensory cues. This model provides a parsimonious explanation of the additive effects of low fidelity cues, as well as the averaging effect of high fidelity cues, previously documented in other Bayesian cue combination psychophysical studies (Young et al., 1993; Gu et al., 2008). Further, our model accurately predicts PEL and provides the means of deriving an appropriate analytical function that can describe behavior. We demonstrate the Bayesian model as a principled approach in estimating PEL.

## Methods

### Experimental design

An experiment can be conducted to measure the effect of multiple visual cues on PEL—we will follow the experimental design from Matin and Li for comparison (Matin and Li, 2000). Subjects are seated upright with their heads stationary on a chin rest, one meter away from a blackened wall with pitched illuminated lines (visual cue). A laser point target is shown on the wall, along the subjects' midsagittal plane. The subject is asked to identify where their eye level is located by repositioning the laser target, without any visual cues present in the room. The PEL of the subject, due to internal stimuli, is recorded. After this initial measurement, a pair of vertical illuminated lines of different lengths are introduced on the wall. The subject is again tasked with lining up the laser target with their eye level. The PEL of the subject is recorded. This would be continuously repeated with the illuminated lines of different pitches and lengths until multiple measurements are collected for each subject. From this description, we apply a Bayesian framework to derive a model of PEL from first principles allowing us to predict the form of PEL expected prior to performing the experiment.

### Gaussian cue combination

**Figure 1:**
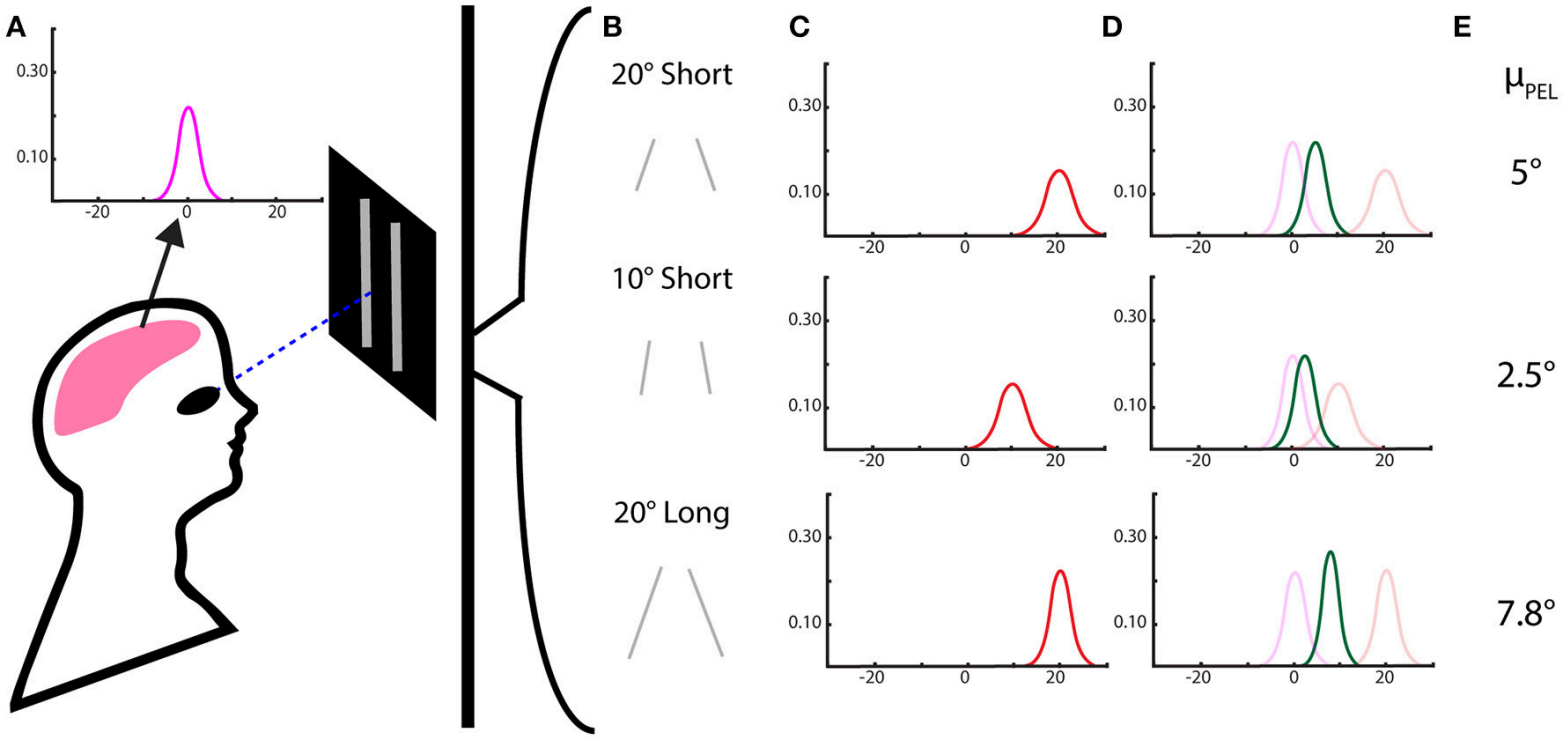
**Perceived eye level using a Bayesian model with Gaussian priors and likelihoods. (A)** The subject's perceived eye level distribution in the dark from proprioceptive and vestibular cues—the “body prior.” **(B)** Two-line stimuli of differing lengths and pitch angles. **(C)** The likelihood for eye level estimation from the 2-line visual stimuli, with means centered on the angle expected from a geometric interpretation and variances dependent on the length of the lines. **(D)** The resulting posterior distribution, the combination of the internal stimuli and the visual stimuli, as determined by Bayes rule. **(E)** The means of the posterior distributions, the expected reported value for perceived eye level, are then calculated, based on the Bayesian model.

In order to use a Bayesian framework, we must define the prior, likelihood, and posterior distributions. Our prior in this experiment is the body-referenced mechanism for PEL estimation—the internal cues are what originally structure our belief about eye level before being presented with stimuli. We approximate this prior as a Gaussian distribution with a mean μ_*b*_ and standard deviation σ_*b*_. There is also the estimate of eye level that comes directly from a geometric interpretation of visual information. In this case, lines can be pitched to give the appearance of a particular tilt from vertical, assuming the observed line is vertical but tilted due to geometric perspective. This purely visual contribution to the perception is μ_*vi*_ for individual visual cues and μ_*v*_ for the combination of visual cues—and similarly σ_*vi*_ and σ_*v*_ for the standard deviations. The posterior distribution, or the effect of *n* visual stimuli and internal cues on PEL, would be the combined effect of the body prior and the visual likelihood, described by μ_*p*_ and σ_*p*_. This posterior distribution would then provide the estimate of the perceived of eye level. These distributions and their final combinations are demonstrated in Figure 1.

As a first step in deriving the equation for PEL in this experimental condition, we first combine the likelihoods of multiple pitched lines. Since we are using Gaussian distributions for our likelihoods, we can achieve this by multiplying them together. The resulting distribution is also Gaussian, with a mean μ_*v*_ and variance $\sigma_{v}^{2}$:

(1) $$\begin{array}{r} \mu_{v\;}=\;\left[\sum_{i=1}^{n}\frac{\mu_{vi}}{\sigma_{vi}^{2}}\right]\sigma_{v}^{2} \end{array}$$

(2) $$\begin{array}{r} \frac{1}{\sigma_{v}^{2}}\;=\;\sum_{i=1}^{n}\frac{1}{\sigma_{vi}^{2}} \end{array}$$

As $\sigma_{v}^{2}$ is in both equations, we can simplify through substitution to obtain the following likelihood estimate based only on visual information. This is the composite likelihood function which will be used in the derivation of μ_*p*_.

(3) $$\begin{array}{r} \mu_{v\;}=\;\left[\sum_{i=1}^{n}\frac{\mu_{vi}}{\sigma_{vi}^{2}}\right]*\frac{1}{\sum_{i=1}^{n}\frac{1}{\sigma_{vi}^{2}}} \end{array}$$

### Estimating variances for the model

To use Gaussian distributions for the priors and likelihoods, two variables are necessary for each stimulus—the mean and variance. The means for the body and individual visual stimuli can be assumed/easily measured and estimated based on geometric interpretation, respectively. However, the variances must be experimentally determined.

**Figure 2:**
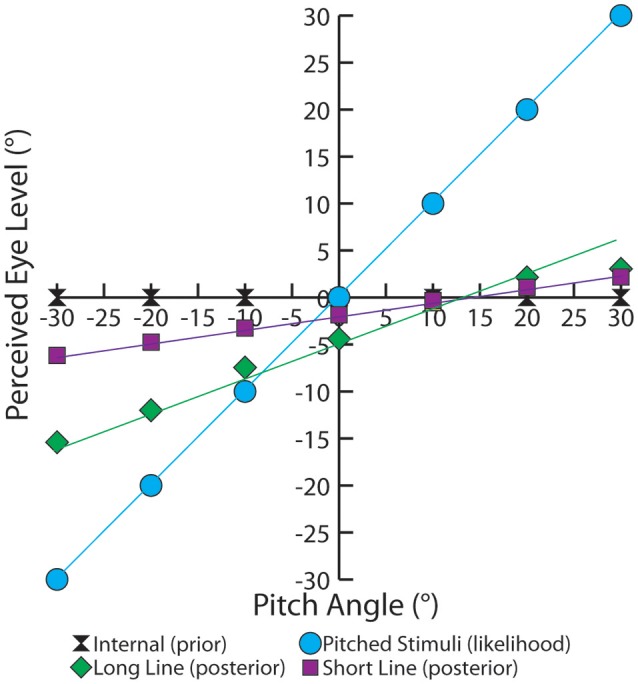
**This graph depicts the experimental trends of average perceived eye level based on pitch**. The prior (black), the body-referenced mechanism, is centered on the true eye level. The likelihood (blue), represents PEL if only the visual information was used. The experimentally measured PEL is shown for single short line (purple) and long line (green). The equation for the short-line regression is y = 0.1415x − 1.869, whereas the equation for the long-line regression is y = 0.3234x − 4.9429. These single line slopes are used to solve for the variances in our model, as shown in Equation (5).

A common method to quantifying the effect of priors and likelihoods in Bayesian analysis is to observe the change in the percept as the mean of the likelihood is altered. When plotted, as done in Figure 2, the slope indicates the relative strength of the visual likelihood compared to the body-based prior. Analytically, this slope is directly related to the ratio of the variances, as shown in Equation (4). Using experimental data of true angle versus perceived angle from Matin and Li this slope, *m*, can be experimentally determined.

(4) $$\begin{array}{r} m=\;\frac{\sigma_{b}^{2}}{\sigma_{b}^{2}+\;\sigma_{v}^{2}} \end{array}$$

With only the two unknown variances in the above equation, having one value will be sufficient for estimating PEL. Fortunately, $\sigma_{b}^{2}$, is readily available experimentally by recording the standard deviation of the PEL estimates when there is no visual information. Using this information, we can then estimate the variance of the combined visual likelihood using Equation (5).

(5) $$\begin{array}{r} \sigma_{v}^{2}=|\frac{\sigma_{b}^{2}}{m}-\sigma_{b}^{2}| \end{array}$$

## Results

### Derivation of the Bayesian model

Proceeding from Equation (3), if line length is constant, all visual stimuli will have equal variance (σ_*vi*_ are equal for all values of i). As such, σ_*vi*_ can be factored out of both summations and $\sum_{i=1}^{n}1$ becomes *n*. Further, the likelihood mean from each line, μ_*vi*_, can be relabeled θ_*i*_ for clarity as the angle is known and does not need to be estimated. This allows us to create a new estimated likelihood mean based on visual cues of the same line length.

(6) $$\begin{array}{r} \mu_{v}\;=\;\left[\;\frac{1}{\sigma_{vi}^{2}}\sum_{i=1}^{n}\theta_{i}\right]\;*\;\frac{1}{\frac{n}{\sigma_{vi}^{2}}} \end{array}$$

We proceed to introduce the prior internal (“body”) mean, μ_*b*_, and variance, $\sigma_{b}^{2}$ (which are equivalent to the mean and variance of PEL in complete darkness). Using the same, multiplication-of-Gaussians approach to find the mean and variance of the posterior, as discussed in the Methods Section, we arrive at the following formulation for the mean PEL.

(7) $$\begin{array}{r} \mu_{p}\;=\;\left[\frac{\mu_{b\;}}{\sigma_{b}^{2}}+\;\frac{1}{\sigma_{vi}^{2}}\sum_{i=1}^{n}\theta_{i}\right]*\frac{1}{\frac{1}{\sigma_{b}^{2}}+\frac{n}{\sigma_{vi}^{2}}} \end{array}$$

This equation can be algebraically rearranged and simplified to:

(8) $$\begin{array}{r} \mu_{p}\;=\;\frac{\mu_{b}}{1+\;\frac{n\;\sigma_{b}^{2}}{\sigma_{vi}^{2}}}+\;\frac{\sum_{i=1}^{n}\theta_{i}}{\frac{\sigma_{vi}^{2}}{\sigma_{b}^{2}}+\;n} \end{array}$$

However, we note that σ_*vi*_ depends on line length, and we would like a function independent of line length if possible. If we assume each additional segment of line length provides an independent sample to establish the slope of the line, we can approximate the relationship between line length and visual variance as an inversely proportional relationship. The validity of this independence assumption is clearly more questionable as line length increases, however, this approach provides a theoretically-justifiable approximation to parametrically estimate behavior when presented with variations in line lengths. Under this assumption, the form of that relationship is then similar to the standard error estimate from a sample mean, $\sigma_{vi}=\sigma_{vl}/\sqrt{\left(l\right)}$, where σ_*vl*_ is independent of line length. The result of this substitution yields:

(9) $$\begin{array}{r} \mu_{p}\;=\;\frac{\mu_{b}}{1+\;\frac{n\sigma_{b}^{2}l}{\sigma_{vl}^{2}}}+\;\frac{\sum_{i=1}^{n}\theta_{i}}{\frac{\sigma_{vl}^{2}}{\sigma_{b}^{2}l}+\;n} \end{array}$$

This is our final model, however, we can make a few substitutions to simplify use in an experimental setting. The first term in Equation (9) can be simplified as a function based on line length, abbreviated *a(l)*, as given in Equation (11). Note, this indicates than an increase in the number of lines or line length should diminishes the value of *a(l)*. Further, $\frac{\sigma_{vl}^{2}}{\sigma_{b}^{2}}$ is composed of constants, so it can be substituted as a constant, *k*, as shown in Equation (12). This yields a simple form of the model to predict average PEL, given in the follow set of equations:

(10) $$\begin{array}{r} \mu_{c}=\;a\left(l\right)+\;\frac{\sum_{i=1}^{n}\theta_{i}}{\frac{k}{l}+n} \end{array}$$

(11) $$\begin{array}{r} a\left(l\right)=\frac{\mu_{b}}{1+\;\frac{nl}{k}} \end{array}$$

(12) $$\begin{array}{r} k=\;\frac{\sigma_{vl}^{2}}{\sigma_{b}^{2}} \end{array}$$

### Predictive ability of the Bayesian model

To test the validity of our derived Bayesian model in experimental circumstances we used data from the experiment outlined in Section Methods, performed by Matin and Li (2001). The experiment was performed with 2 line lengths (short 12° and long 64°) with pitches from −30 to 30° in 10° increments. According the Bayesian model as pitch angle (θ_*i*_) increases, the mean PEL increases as well. However, PEL will never exceed the pitch angle. This is demonstrated in Figure 2. As such, PEL lies between estimates from internal cues (prior) and the pitch angle. Similarly, as the strength (line length) of a visual input increases, so does the influence of the visual stimuli on the perception of eye level, as PEL moves away from the prior. Both of these phenomena are demonstrated experimentally (Matin and Li, 2000, 2001).

**Figure 3:**
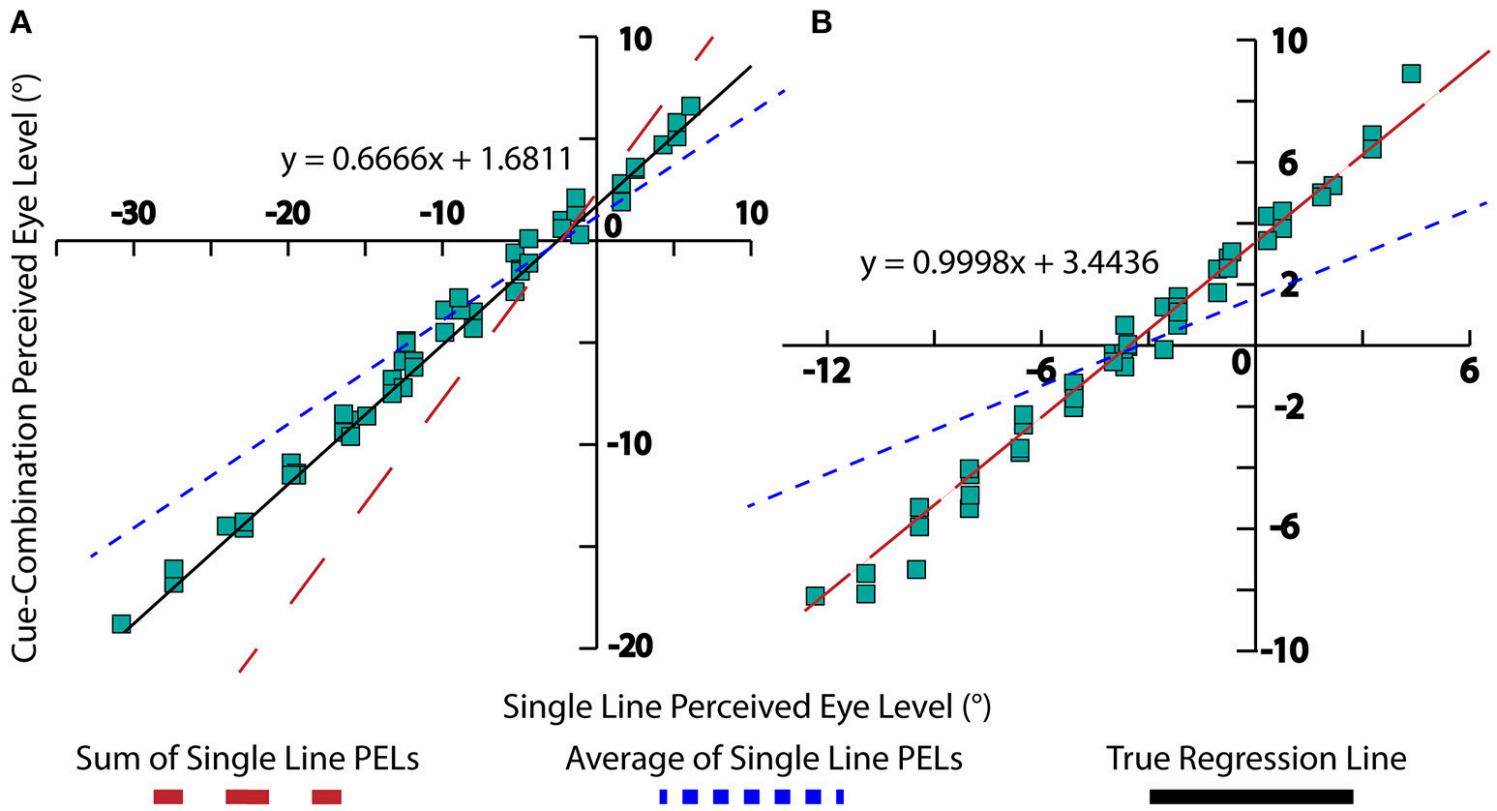
**Experimental data, collected by Matin and Li**. A linear regression for the data is displayed (black), as well as lines displaying the summation of single-line PELs (red) and averaging of single-line PELs (blue). **(A)** This depicts long-line experimental data with *m* = 0.666, signifying a near-averaging relationship between high-fidelity cues (blue dotted line). **(B)** This chart depicts short-line experimental data with *m* = 0.9998, signifying a summative relationship between low-fidelity cues (red dotted line).

The interpretation of multiple visual cues demonstrates an interesting perceptual trend. When two separate visual cues are observed independently, two distinct percepts of PEL are obtained. When the cues are given together, the resulting PEL appears to fall in two distinct regimes. Weak cues (e.g., short lines) PELs are additive, while strong cues (e.g., long lines) are averaging. This is a fact that have been observed and explained in previous psychophysical studies (Young et al., 1993; Gu et al., 2008) as well as neural cue integration (Fetsch et al., 2013). This was also demonstrated experimentally, long-line stimuli almost averaged in their influence on PEL and short-line stimuli appearing to be summed in the determination of PEL (Matin and Li, 1999, 2000). Figure 3 is an adaptation of the result from Matin and Li demonstrating this phenomenon. One can also observe from Equation (10) that as line length increases, the denominator tends toward “*n*” thus approaching an averaging effect, while shorter line lengths lead to the constant “*k*” dominating the denominator which leads to a summation effect. The compensatory effect of *a(l)* should be minimal given the tendency of the body prior to be near 0. In this way, this observed combination effect can be seen analytically in our model.

**Figure 4:**
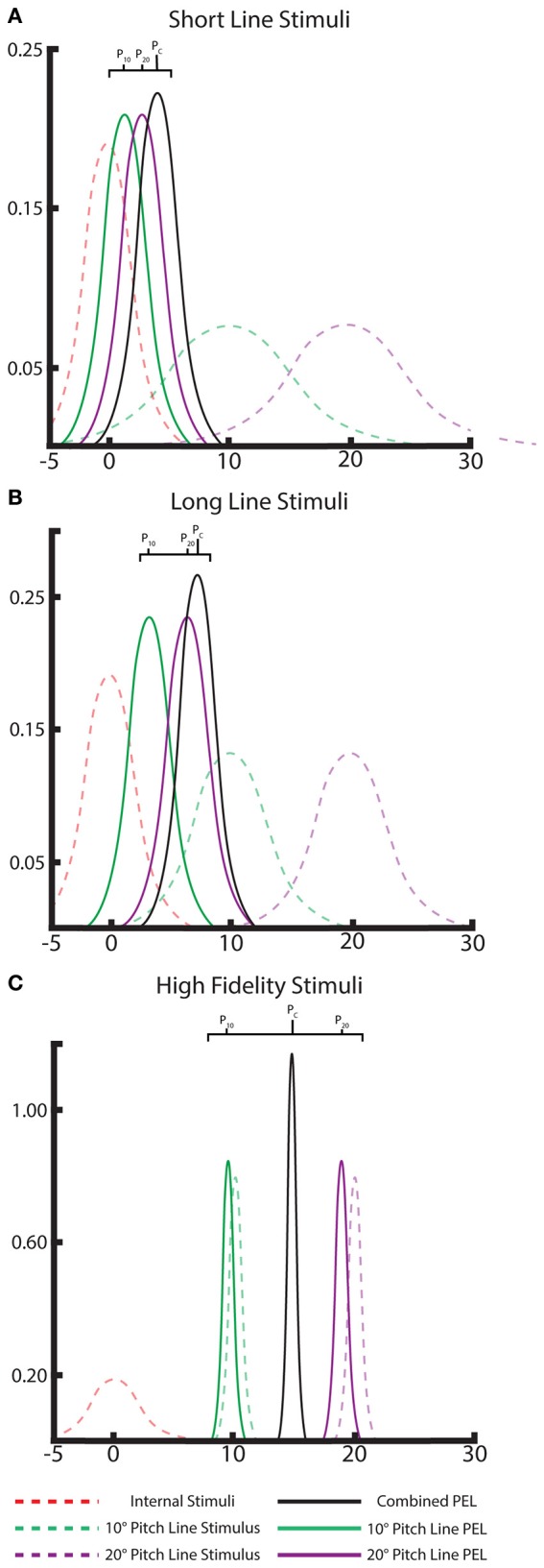
**A Bayesian interpretation of the “averaging” and “additive” effects of multiple cues in PEL**. Gaussian distributions of the internal priors (dashed, red), likelihood of the individual visual lines (dashed, green and purple), and the single-line posterior distributions/PELs for each visual cue (solid, green and purple). The location of these PEL posterior means can be compared to the combined-cue posterior (black) to note the averaging or additive effect of combining multiple cues. This is shown for both long-line cues **(A)** and short-line cues **(B)** and displays the “nearly averaging” and additive effects, respectively, as seen in the data. Ideal, high-fidelity stimuli are shown as well **(C)** demonstrating a clear averaging effect.

Note, this additive/averaging effect in cue combination is well understood in Bayesian literature. Our model also exhibits these effects straightforwardly by adjusting the likelihood variances based on line length. Weaker, short line stimuli lead to shallow, high-variance likelihoods which individually only marginally move the posterior from the body prior, but together the combined posterior moves farther than any one alone (“additive”). With the strong, low-variance likelihoods from long line stimuli, the posteriors are dominated by the likelihoods, leading to a combined posterior between the likelihoods when presented with two strong long line stimuli (“averaging”). The visual demonstration of this effect using the Bayesian framework is shown in Figure 4. The Bayesian approach successfully explains the effects observed with multiple cues.

### Comparison to previous model

From experimental results and fitting the behavioral data, a model was previously developed to explain the effects of multiple lines on the perception of eye level (Matin and Li, 2001).

Bayesian Model:

(13) $$\begin{array}{r} PEL=\mu_{p}=\;a\left(l\right)+\;\frac{\sum_{i=1}^{n}\theta_{i}}{\frac{k}{l}+n} \end{array}$$

Matin and Li Model:

(14) $$\begin{array}{r} PEL=\;a+\;\frac{k_{1}\sum_{i=1}^{n}\theta_{i}}{\frac{k_{2}}{l}+n} \end{array}$$

The Matin and Li model is very similar to our model. The notable difference between the previous model and the Bayesian model is the existence of *k*_1_ in the Matin and Li model. Another small difference remains, as *a* is defined as a constant in the Matin and Li model, whereas it is derived as a function dependent on line length in our model. Our framework also allows the model to be derived for various different scenarios, such as for multiple lines of different lengths, as well as has the ability to be extended to other studies, perhaps even unrelated to PEL as studied here.

From experimental results shown in Figure 2 we calculated variance estimates for single line cues from a linear fit of the data. These results are presented in Table 1. With the prior and likelihood distributions' variance estimates, all necessary values are known for a complete Bayesian model. The fitted constants for Matin and Li's behavioral model (Equation 14) are displayed in Table 2. For each model we predicted the mean PEL given the experimental condition. Using the standard error in estimation we measured the accuracy of our predictions, displayed in Table 3. Both models are accurate (no standard errors over 3°) with the Matin and Li model slightly more accurate for long-line stimuli and the Bayesian model performing better with short-line stimuli. This is fitting with the independence assumption for how line segments contribute to variance estimates, which suggested the Bayesian model may not hold as well for longer line stimuli.

**Table 1:** **Standard deviation and mean estimates for single-line inputs calculated from experimental data as discussed in Methods**.

**Bayesian model parameters**	
**Parameter**	**Value**
Internal (prior) std dev	±2.08
Internal (prior) mean	−0.622
Long line likelihood std dev (σ_*vi*_)	±3.01
Short line likelihood std dev (σ_*vi*_)	±5.12

**Table 2:** **Parameters obtained from previous work directly fitting to behavioral data**.

**Matin and Li model parameters**		
**Constant**	**Short line values**	**Long line values**
a	−0.29	−4.61
*k_1_*	0.51	0.51
*k_2_*	19.44	19.44

**Table 3:** **Using variance estimates from Table 1 in our Bayesian model and constant values from Table 2 in the Matin and Li Model, we predict the effect of two short or long pitched-from-vertical line stimuli on perceived eye level**.

**Model**	**Short-12° SEE**	**Long-64° SEE**
Bayesian	±0.78	±4.81
Matin and Li	±1.63	±2.57

*The predicted means were close to those observed experimentally, signified by a low Standard Error of Estimation (SEE). Similar to the Matin and Li model, our predictions were more accurate for short-line stimuli than long-line stimuli*.

## Discussion

Our Bayesian framework successfully derived a model to predict PEL from first principles without the need for experimental data. Whereas, the Matin and Li derived equation could only take into account *n* visual stimuli of the same length, our framework can be extended analytically to take into account *n* visual stimuli of varying lengths, all from the same basic principles demonstrated in this manuscript. The simplicity of the Bayesian framework is also an indication of its latent power for prediction. This framework can be analytically derived and expanded to multiple studies in a straightforward way, including multiple lines of varying lengths, non-line visual stimuli, and manipulations of the body prior for PEL. The same tools can be applied to enable a straightforward interpretation for combining these, or additional stimuli, to estimate the percept.

It is important to stress that this Bayesian interpretation does not preclude a more detailed, biophysical explanation. The approach explains the computational principles likely involved, but not how they are implemented, consistent with David Marr's levels of analyses (Marr, 1982). Marr categorizes all models into three levels: the computational level, the algorithmic level and the implementation level. Here, we only address the first level, which constrains but does not conflict with other potential models that explain this behavior. Matin and Li's experimentally justified creation of Equation (13) provides a succinct, high-level summary of behavior; however, it should be apparent that such fitting to the data may explain “what” is occurring, but a Bayesian derivation adds a clearer picture of “why” such an equation fits the data as presented. Again, this Bayesian interpretation does not preclude other lower-level interpretations. For example, Matin and Li in 2001 proposed a neurophysiological model to explain this behavior. This is a separate level of analysis as the Bayesian approach and should not be considered in conflict with this, or other, potential algorithmic or implementation-level explanations of observations.

Our Bayesian PEL model adds to the growing repertoire of Bayesian perceptual papers addressing a wide range of perceptual topics. Bayesian principles have explained visual illusions in end-point occluded or low-contrast rhombus motion (Weiss et al., 2002). Bayesian inference can predict the effect of visual and vestibular cues in the determination of heading (Butler et al., 2010). Bayesian integration has also successfully modeled the effect of visual and auditory signals on spatial localization (Battaglia et al., 2003). Our PEL Bayesian analysis and Bayesian framework adds to this body of knowledge further demonstrating the power of Bayesian techniques in perceptual studies.

We have successfully demonstrated a Bayesian framework to model cue combination in a perceptual study of PEL. Using the approach we derived a model for PEL from first principles which matched the experimentally determined behavior demonstrated by researchers Matin and Li. Further, this model explains trends and phenomena associated with PEL, providing a straightforward, parsimonious explanation of the “summing” and “averaging” effects on PEL when combining weak and strong visual cues. Most importantly, the Bayesian approach is more amenable to incorporate different experimental conditions, such as varied line lengths or additional stimulus types. This framework is more parsimonious, and subsequently more powerful, further enabling other perceptual studies.

## Author contributions

EO and MA designed the study and performed the necessary derivations. EO extracted the behavioral data, performed the analyses, and created the initial draft of the manuscript. RP aided in the initial analysis steps. LK confirmed the results and completed the manuscript.

## Funding

MA was funded through startup costs for the Pervasive and Ambient Computing (PAC) lab at Loyola University Chicago. The work of author LK was funded by NIH Grant R01NS063399 to Konrad Kording.

### Conflict of interest statement

The authors declare that the research was conducted in the absence of any commercial or financial relationships that could be construed as a potential conflict of interest.
